# Anti-inflammatory and wound healing activities of calophyllolide isolated from *Calophyllum inophyllum* Linn

**DOI:** 10.1371/journal.pone.0185674

**Published:** 2017-10-11

**Authors:** Van-Linh Nguyen, Cong-Tri Truong, Binh Cao Quan Nguyen, Thanh-Niem Van Vo, Trong-Thuc Dao, Van-Dan Nguyen, Dieu-Thuong Thi Trinh, Hieu Kim Huynh, Chi-Bao Bui

**Affiliations:** 1 Biotechnology Research and Development Institute, Can Tho University, Can Tho City, Viet Nam; 2 Laboratory of Cutaneous Research, Center for Molecular Biomedicine, University of Medicine and Pharmacy at Ho Chi Minh City, Ho Chi Minh City, Viet Nam; 3 Nanoproduct Laboratory, Faculty of Pharmacy, University of Medicine and Pharmacy at Ho Chi Minh City, Ho Chi Minh City, Viet Nam; 4 Genetics and Plant Breeding Department, CuuLong Delta Rice Research Institute, Can Tho City, Viet Nam; 5 PAK Research Center, Okinawa, Okinawa, Japan; 6 Faculty of Traditional Medicine, University of Medicine and Pharmacy at Ho Chi Minh City, Ho Chi Minh City, Viet Nam; Universitatsklinikum Hamburg-Eppendorf, GERMANY

## Abstract

Due to the high-cost and limitations of current wound healing treatments, the search for alternative approaches or drugs, particularly from medicinal plants, is of key importance. In this study, we report anti-inflammatory and wound healing activities of the major calophyllolide (CP) compound isolated from *Calophyllum inophyllum* Linn. The results showed that CP had no effect on HaCaT cell viability over a range of concentrations. CP reduced fibrosis formation and effectively promoted wound closure in mouse model without causing body weight loss. The underlying molecular mechanisms of wound repair by CP was investigated. CP markedly reduced MPO activity, and increased M2 macrophage skewing, as shown by up-regulation of M2-related gene expression, which is beneficial to the wound healing process. CP treatment prevented a prolonged inflammatory process by down-regulation of the pro-inflammatory cytokines—IL-1β, IL-6, TNF-α, but up-regulation of the anti-inflammatory cytokine, IL-10. This study is the first to indicate a plausible role for CP in accelerating the process of wound healing through anti-inflammatory activity mechanisms, namely, by regulation of inflammatory cytokines, reduction in MPO, and switching of macrophages to an M2 phenotype. These findings may enable the utilization of CP as a potent therapeutic for cutaneous wound healing.

## Introduction

Skin functions as a protective barrier against physical damage, fluid loss, and the invasion of toxic substances [1,2]. Cutaneous wounds are physical injuries resulting in opening or breaking of the skin, thereby causing a pertubation in the normal skin anatomy and function [3,4]. When the skin is injuried, platelets will initiate a hemostatic reaction to prevent the loss of the blood at the wound. This reaction is characterized by vascular constriction, platelet aggregation and degranulation, coagulation, and finally formation of a fibrin clot. This clot further induces migration of inflammatory cells to the injuried site. After this phase, the wound healing process proceeds by way of three major overlapping events including an inflammation phase, cell proliferation/formation of granulation tissue phase, and remodeling/scar formation phase with all events requiring the interplay of many cell types [5,6]. Firstly, inflammatory cells (mainly neutrophils and monocytes) migrate to the site of injury to carry out phagocytic and antimicrobial functions. In the meantime, resident macrophages kill any invading microbes, remove tissue debris, and stimulate the proliferation of fibroblasts and the epithelial cells at the wound site [7,8]. The proliferative phase occurs simultaneous events to form granulation tissue, establish an approriate blood supply (neovascularization), reinforce the injuried tissues (fibroplasia), and synthesize extracellular matrix [7–9]. The remodeling phase is the final stage of the wound healing process, consisting of wound closure, collagen synthesis, reepithelialization, and scar tissue formation, which can take up two years or can continue indefinitely [7–9]. Consequently, if the inflammatory response is elongated or exacerbated, it leads to a delay in the subsequent phases of proper wound healing and scar formation. There is strong evidence that pro-inflammatory cytokines (IL-1β, IL-6, and TNF-α) released by macrophages are involved in the up-regulation of inflammatory reactions, and in the process of pathological pain [10] while the wound healing is accelerated by appropriate temporal down-regulation of pro-inflammatory cytokine levels [11]. Production of anti-inflammatory agents to suppress pro-inflammatory cytokines are thus required to reduce this inflammatory phase.

*Calophyllum inophyllum* Linn. (*C*. *inophyllum*, Family: Guttiferae) has been widely used in folk medicine for treating a variety of diseases [12]. In Vietnam, *C*. *inophyllum* oil has been used as a traditional medicine to treat burns, skin-related and rheumatic diseases, and insomnia [13]. Extensive phytochemical analyses of this species has shown that *C*. *inophyllum* possesses a source of diverse bioactive compounds including coumarins, xanthones, flavonoids, steroids, and triterpenoids [14,15]. Calophyllolide (CP), a major constituent isolated from *C*. *inophyllum*, has been reported to have anti-inflammatory, anti-coagulant, anti-microbial, and even anti-cancer activities [16–19]. CP also shows potent anti-inflammatory activity in carrageenin-induced edema in albino rats although its underlying molecular mechanisms remains unclear [15]. Since CP exhibits anti-inflammatory activity, it is likely that this compound could promote wound healing. Herein, we tested whether CP could promote wound healing in a murine mouse model. Furthermore, we measured protein levels of cytokines including tumor necrosis factor-α (TNF-α), interleukin (IL)−1β, IL-6, and IL-10, as well as indicators of M2 macrophage activation in an attempt to understand the underlying molecular mechanism of potential wound repair by CP.

## Materials and methods

### Isolation of calophyllolide (CP) compound from *Calophyllum inophyllum* seeds

The *C*. *inophyllum* sample used in this study was collected at Chau Thanh District, Soc Trang Province, Vietnam, at 9°42'15.2"N and 105°54'20.6"E. This sample was granted and approved by the Soc Trang Province Folk Medicine Association (Viet Nam), and then classified by the Department of Medicinal Plants, Faculty of Traditional Medicine, University of Medicine and Pharmacy HoChiMinh City. Calophyllolide (CP) compound was isolated according to a previously described method [12]. Fresh seeds (200 g) were ground by a rotor mill (ZM 200, Retsch, Haan, Germany) and extracted with 100% EtOH (850 mL) by continuous shaking at 60°C for 10 min. The mixture was filtered, and the resulting filtrate was concentrated by evaporator under reduced pressure to give a crude extract (18 g). The dried crude extract was dispensed in distilled water and partitioned with an equal volume of ethyl acetate (EtOAc). The EtOAc-soluble fraction was subjected to column chromatography and eluted with the solvent mixture of *n-*hexane:EtOAc (6:1). The aliquots from column chromatography were subjected to Sephadex LH-20 column chromatography (Amersham Bioscience, Uppsala, Switzerland) and eluted with 100% MeOH, and the resulting fraction was analyzed by reverse-phase (RP) high performance liquid chromatography (HPLC). Purified CP was collected at 233 nm using a Sunfire Waters C_18_-column (Saint-Quentin en Yvelines, France) (150 mm × 4.6 mm i.d.) with a particle size of 3.5 μm on a Varian LC-920 system (Agilent technologies, Les Ulis, France) equipped with quaternary pumps and an UV-Vis DAD. The mobile phase used was water (solvent A) and acetonitrile (solvent B), and the flow rate was at 1 mL/min. The gradient elution was performed as follows: 1–40 min for 90% solvent B and 40–60 min for 50% solvent B. The pure CP obtained from Danapha Co. (Danang, Viet Nam) was used as an external standard in the HPLC analysis. Identification of CP was carried out by matching the retention time of the major peak with those of the standard compound. NMR and mass spectra data of isolated calophyllolide was in agreement with those of previously reported one [20]. ^1^H-NMR (400 MHz, CDCl_3_): *δ* 0.95 (s, 3H), 1.87 (d, *J* = 6.6 Hz, 3H), 1.99 (s, 3H), 3.74 (s, 3H), 5.46 (d, *J* = 9.6 Hz, 1H), 6.00 (s, 1H), 6.43 (d, *J* = 9.6 Hz, 1H), 6.54 (q, *J* = 6.6 Hz, 1H), 7.22 (m, 2H), 7.36 (m, 3H). ^13^C-NMR *δ* 10.76, 15.22, 26.91, 63.04, 105.67, 110.77, 114.31, 115.12, 116.01, 127.31, 127.52, 127.79, 129.03, 139.59, 140.01, 144.20, 149.62, 151.75, 152.05, 154.99, 155.91, 159.50, 194.30. ESI-MS *m/z* 417.3 [M+H]^+^.

### Cell culture

The immortalized human keratinocyte cell line (HaCaT) and RAW264.7 were kindly provided from Dr. Mike Philpott (Blizard Institute, London, UK) [21] and Dr. Binh Nguyen (PAK Research Center, Okinawa, Japan), respectively. Cells were cultured in medium containing Dulbecco's modified eagle's medium (D-MEM), 100 U/mL penicillin- streptomycin (Life Technologies, California, USA), and 10% fetal bovine serum (FBS) (Gibco, Thermo Fisher Scientific, Massachusetts, US).

### Cell viability assay

The effect of calophyllolide (CP) on HaCaT and RAW264.7 (American Type Culture Collection, USA) cell viability was tested by MTT assay. Cells were seeded at a density of 1 × 10^4^ cells/well in 96-well plates (Corning, New York, USA) for 24 h, and then treated with different concentrations of calophyllolide (CP) ranging from 10–1000 ng/mL for 24 h. Cell viability was assessed by MTT assay (Promega) as previously described [22]. The absorbance was read at 570 nm using microplate autoreader (Bio-Tek Instruments, CA, USA).

### Experimental animals

Eight-week-old *Mus musculus* var. Albino male mice (weighing 30 ± 2 g) were obtained from Pasteur Institute (Vietnam Pasteur Institute, HoChiMinh city, Viet Nam). Animals were maintained in an animal house under a12 h light:12 h darkness cycle, 50–60% humidity. Animals were given free access to commercial chow (PCR Corp., Cantho, Viet Nam) and distilled water. All animal experiments in this study were approved by the Animal Ethical Committees and Cares at Center for Molecular Biomedicine, University of Medicine and Pharmacy at +HoChiMinh City (CMB-AS-2015-01).

### Incision wound creation and topical treatment

Mice were divided into four groups and treated as follows: (1) without wounding (control), (2) PBS treated (vehicle), (3) povidone-iodine treated (PI), and (4) CP treated. Five mice per group were anesthetized with an intramuscular injection of Ketamine (2 mg/kg body weight) (Solupharm, Germany) and Zoletil (50 mg/kg body weight) (Virbac Laboratoires, Carros, France). The backs of the mice were shaved with an Oster Mark II animal clipper (Sunbeam-Oster, Fort Lauderdale, Florida), and after disinfection with 70% ethanol, one 2.5 cm midline dorsal incision was made on the back through the epidermis, dermis, and subcutaneous tissue layers without injury of the fascia. The skin incision was immediately closed with Carelon (CPT Sutures Corp., HoChiMinh, Vietnam). The sutures were removed at day 5 after skin incision. The treatment consisted of a topical application of 0.5 mL compound (6 mg/animal for CP, 100 mg/animal for PI) on the wound area once daily for 14 days. At each specified time point, mice in each group were sacrificed by CO_2_ inhalation. Cutaneous inflammation was induced by topical application of sodium lauryl sulfate (SLS) (3 mg/mL in distilled water) (Sigma Aldrich, St. Louis, MO). The skin wound samples from each animal were used for biochemical analysis and histological observation.

### Histological and quantitative analyses of the cutaneous wound healing rates

Ten mice per group were sacrificed at days 1, 5, 7 and 14 post-wounding. Skin and spleens were isolated and fixed in 3.7% para-formaldehyde (PFA) (Sigma-Aldrich, St. Louis, MO) and 8% sucrose (Sigma-Aldrich, St. Louis, MO) for 24 h. Sections (4.5 μm thick) were prepared using the Tissue-Tek Cryo3 Plus Microtome/Cryostat (Sakura Finetek Ltd., Tokyo, Japan). For overall observation, hematoxylin and eosin (HE) (Sigma-Aldrich, St. Louis, MO) staining was carried out as per manufacturer’s instructions. For collagen observation, trichrome staining was performed using the NovaUltra™ Masson Trichrome Stain Kit (IHC World, Woodstock, MD). All slides were scanned using the Ventanai Scan Coreo (Ventana Medical System Inc., CA, USA). For quantitative analysis of wound closures, the wound area was assessed as previously described [23]. Briefly, after skin excision, the residual wound area was traced daily until day 14, and pixels of the traced area were analyzed by ImageJ (Software 1.48q, Rayne Rasband, National Institutes of Health, USA). Wound area analysis was blinded, and wound area percentage was calculated by the following formula:

Woundarea(%)=[Area(dayN)/Area(day0)]×100

where Area (day 0) is the initial wound area at day 0, and Area (day N) is the area on day N after wounding.

### Blood and spleen sampling

At assigned time points, ten animals were sacrificed, and blood samples obtained from the carotid artery. Spleens of animal were obtained and weighed. The spleen index was determined as the ratio of spleen to animal body weight (mg/g). Spleens were dissociated, and splenocyte cells were counted by trypan blue staining on countess cell counting chamber slides (Invitrogen, Life Technology). Blood samples were left to coagulate and then centrifuged. Clear non-hemolyzed serum was stored at -20°C.

### Myeloperoxidase assay (MPO)

Tissue myeloperoxidase (MPO) activity was examined using a MPO detection kit (Cell Technology, Mountain View, CA) according to manufacturer's instructions. In brief, skin biopsies were cut into small pieces, and homogenized at 20,000 rpm in 1X assay buffer (T25 ULTRA-TURRAX homogenizer, IKA, Japan). The homogenized tissues were centrifuged at 4,000 rpm at 4°C for 15 min, and the proteinaceous pellets were solubilized in 1X assay buffer containing 0.5% HTA-Br (Hexadecyltrimethylammonium bromide, Sigma). The samples were then homogenized again, sonicated using the Sonic Dismembrator (Fisher Scientific, Pittsburgh, PA) for 30 seconds, and subjected to two cycles of freezing and thawing. 50 μL of each supernatant was transferred into each well of a fluorescent 96-well plate, and subsequently 50 μL of reaction cocktail (detection reagent and hydrogen peroxide in 1X assay buffer) was added. Standard curves were created by serial dilution of a MPO standard. After 30 min incubation, MPO activity was measured at excitation 530 nm and emission 590 nm by microplate autoreader (Bio-Tek Instruments, CA, USA). The experiments were carried out in triplicates.

### Collagen (Sircol) assay

The soluble collagen in wound sites was measured by colorimetrical Sircol Collagen Assay kit (Biocolor Ltd., County Antrim, UK). The samples were homogenized in lysis buffer (100 mM potassium phosphate, 0.3% Triton X-100, pH 7.4), and the debris was removed by centrifugation at 12,000 rpm for 10 min. The collagen content was measured as μg collagen per gram of total protein following the manufacturer’s instructions.

### Enzyme-linked immunosorbent assay (ELISA) for cytokines

TNF-α, IL-10 (BioLegend, San Diego, CA), IL-6, and IL-1β (Thermo Scientific, Seoul, Korea) cytokine expression in the serum was measured by ELISA kit following manufacturer's instructions (RnD Systems, Minneapolis, USA). All reagents, standard dilutions, and samples were prepared as directed. 100 μL and 50 μL of calibrator diluent were added to non-specific binding and zero standard (B_0_) wells, respectively. The remaining wells received 50 μL of the standard, control, or sample. Next, 50 μL of the secondary antibody solution was added to each well, followed by adding 100 μL of conjugate after 2 h. The wells were then incubated with 100 μL of substrate solution. The reaction was stopped when the color of the solution turned blue. The optical density was measured at 450 nm using the Spectro UV-VIS Dual Beam (Labomed, Inc.).

### Reverse transcription and quantitative real time-polymerase chain reaction (qRT-PCR)

Macrophage (M1/M2)-related gene/marker expression was measured by quantitative real time-polymerase chain reaction (qRT-PCR). Total RNA from control and SLS-treated mouse skin was first reverse-transcribed into cDNA by using the PrimeScript™ 1st strand cDNA Synthesis Kit (Cat. #6110A, Takara Bio Inc., Shiga, Japan). mRNA for CD14 and CD127 (markers for M1 macrophage), and CD163 and CD206 (markers for M2 macrophage) was measured by qRT-PCR with SYBR Green PCR Master Mix (Applied Biosystems, Foster City, CA, USA) using designed forward and reverse primers as listed in S1 Table. qRT-PCR reaction was performed in Eppendorf^TM^ Mastercycler^TM^ pro PCR System (Fisher Scientific, Hamburg, Germany) under the following reaction conditions: 3 min at 95°C, then 40 cycles of 20 sec at 95°C, 1 min at 55°C, and 30 sec at 72°C. The housekeeping glyceraldehyde-3-phosphate dehydrogenase (GAPDH) gene was used as the standard, and cycle threshold (CT) values were converted to fold change by using the 2^−ΔΔCT^ formula [24].

### Statistical analysis

Data were analyzed using GraphPad Prism 5.0 (La Jolla, CA). Statistical differences between various means were evaluated by one-way ANOVA and Tukey’s post-hoc test. Mann–Whitney U test was used to measure histopathological analysis. All experimental data were presented as the mean ± standard deviation (SD) of three independent experiments. *P*-values < 0.05 were interpreted as statistically significant.

## Results

### Identification of calophyllolide (CP) isolated from *Calophyllum inophyllum* seeds

**Fig 1:**
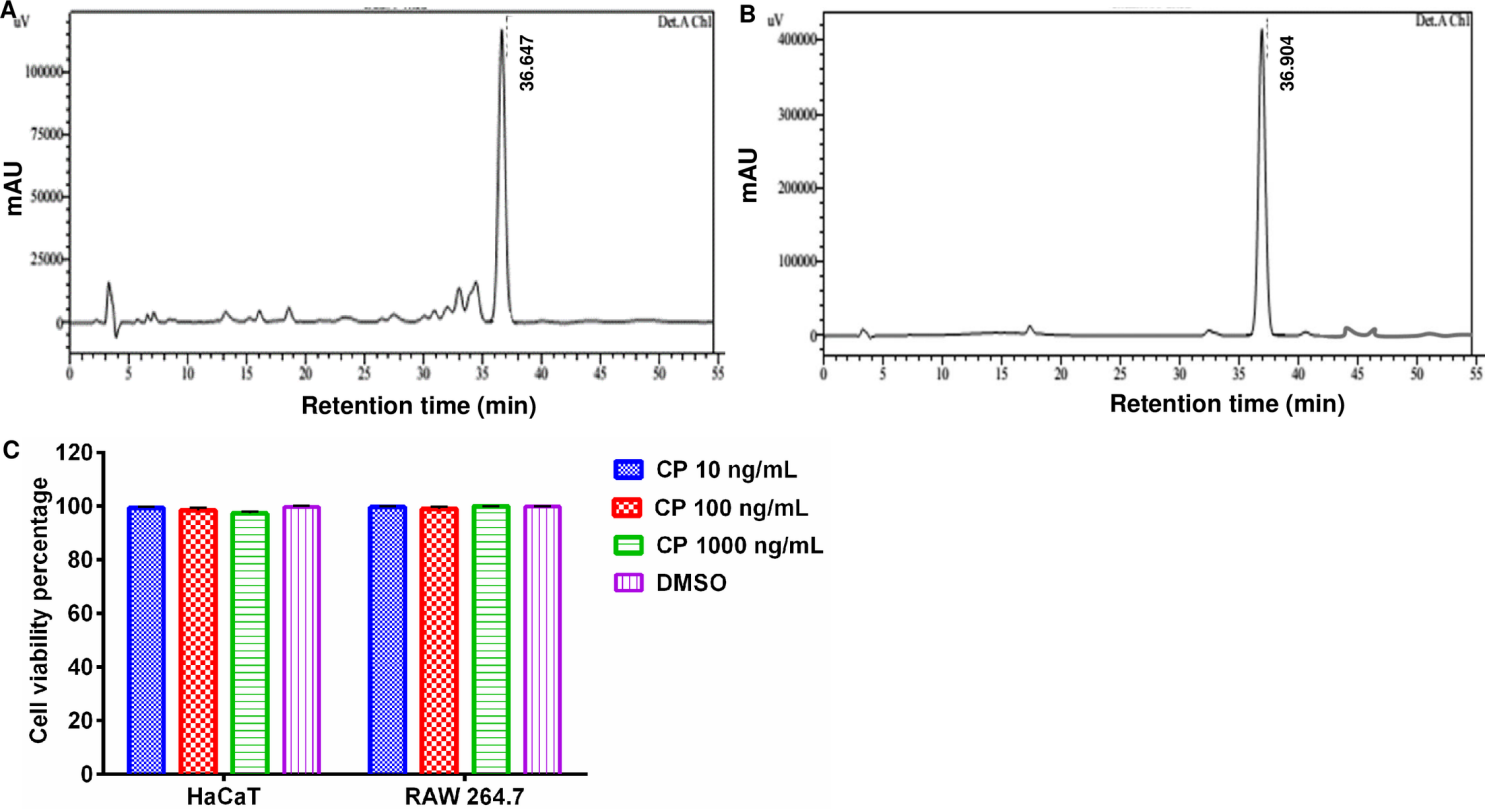
Effect of calophyllolide on HaCaT and RAW264.7 cell viability. HPLC chromatograms of the isolated calophyollide **(A)** and standard control **(B)**. This compound was recorded at 233 nm, and its retention time is 36.6 min. **(C)** No effect of CP on the viability of both HaCaT and murine macrophage RAW264.7 cells after 24 h treatment.

As shown in Fig 1A, calophyllolide (CP) isolated was identified by matching its retention time with those of standard calophyllolide (Fig 1B). The retention time of this compound was 36.6 min. The major peak of CP compound was detected at 233 nm wavelength. NMR spectra and MS data of isolated CP was in good agreement with those of previously reported one (S1–S3 Figs) [20]. The isolated calophyllolide had 91% purity level.

### Effect of calophyllolide (CP) on HaCaT cell viability

In order to test whether isolated CP had any effect on HaCaT and RAW264.7 cell growth, the cells were cultured in the absence or presence of this compound at various concentrations ranging from 10–1000 ng/mL before cell viability was assessed by MTT assay. The results showed that there was no significant effect of CP on HaCaT and RAW264.7 cell viability after 24 h treament at indicated concentrations (Fig 1C).

### Acceleration of faster wound closure by calophyllolide (CP)

**Fig 2:**
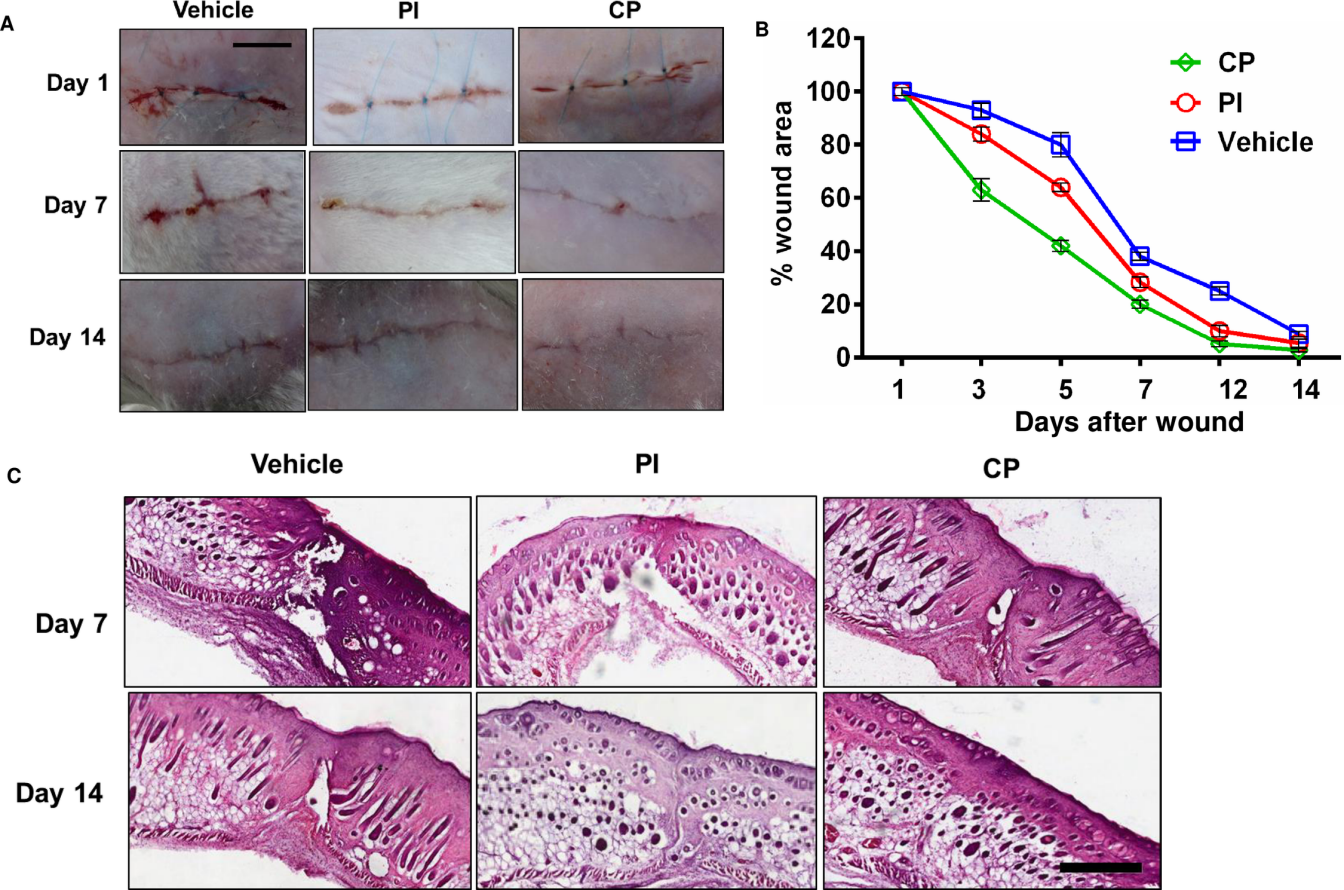
The enhancement of wound closure by calophyllolide. Mice were daily treated with CP (6 mg/animal) and PI (100 mg/animal) until enthanasia. **(A)** Process of surgical wound healing in CP-treated group versus vehicle and PI-treated group, scale bar = 1cm (five animals per group). **(B)** The wound area (%) in the three treatment groups from day 1 to day 14 (n = 3–4 animals per group per experiment). **(C)** HE staining of cutaneous wound healing at day 7 and day 14 post-operation. Arrows indicate the wound sites, scale bar = 500 μm.

Next we tested whether CP could promote cutaneous wound healing in a mouse model. We created a surgical wound and then treated topically with PBS (vehicle), povidone-iodine (PI) or calophyllolide (CP). Unwounded mice were included as a control group. As shown in Fig 2A, CP effectively promoted the closure of the wound faster than vehicle and PI at day 7 and 14 post-treatment. CP also increased the recovery of the wound area in comparison to vehicle and PI. CP promoted the closure of the wound area by around 80% and 97% at day 7 and day 14 post-treatment whereas PI promoted closure of the wound area by approximately 71% and 93% at day 7 and day 14 post-treatment, respectively (Fig 2B and Table 1). Observational and HE staining analyses were carried out at day 7 and 14 post-wounding. On day 7, a reduced blood clot covering wound was observed in the PI- and CP-treated group. HE examination of the scars at day 14 post-wounding showed that the CP-treated group had reduced fibrosis and faster closure of wound when compared to vehicle- and PI-treated groups (Fig 2C). In addition, complete revovery of the epidermal and dermal layers were observed in the CP-treated group at day 14. However, CP did not significantly alter proliferation and apoptosis during the early onset of wound healing, as shown by BrdU and TUNEL analyses (S4A and S4B Fig, respectively). Moreover, body weight was not affected at attested time points (S2 Table), therefore CP in fact did the faster wound closure without causing body weight loss.

**Table 1:** Effect of calophyllolide (CP) on acceleration of wound closure.

	Wound area (%)		
	Vehicle	PI	CP
Day 3	94.17 ± 0.76	85.50 ± 2.18^###^	65.50 ± 2.3 ***
Day 5	85.67 ± 4.04	66.17 ± 2.47^##^	51.67 ± 4.93***
Day 7	39.67 ± 1.89	29.83 ± 0.76^###^	21.17 ± 1.26***
Day 12	27.00 ± 1.50	10.67 ± 2.23	6.0 ± 1.73***
Day 14	9.67 ± 2.51	6.33 ± 1.53	2.83 ± 1.04**

Data are represented as mean ± SD.

^##^*P*< 0.01

^###^*P*< 0.001 compared between CP-treated group with PI-treated group

**P<0.01

***P< 0.001 compared between CP-treated group with vehicle group.

n = 3–4 animals per group per experiment.

**Fig 3:**
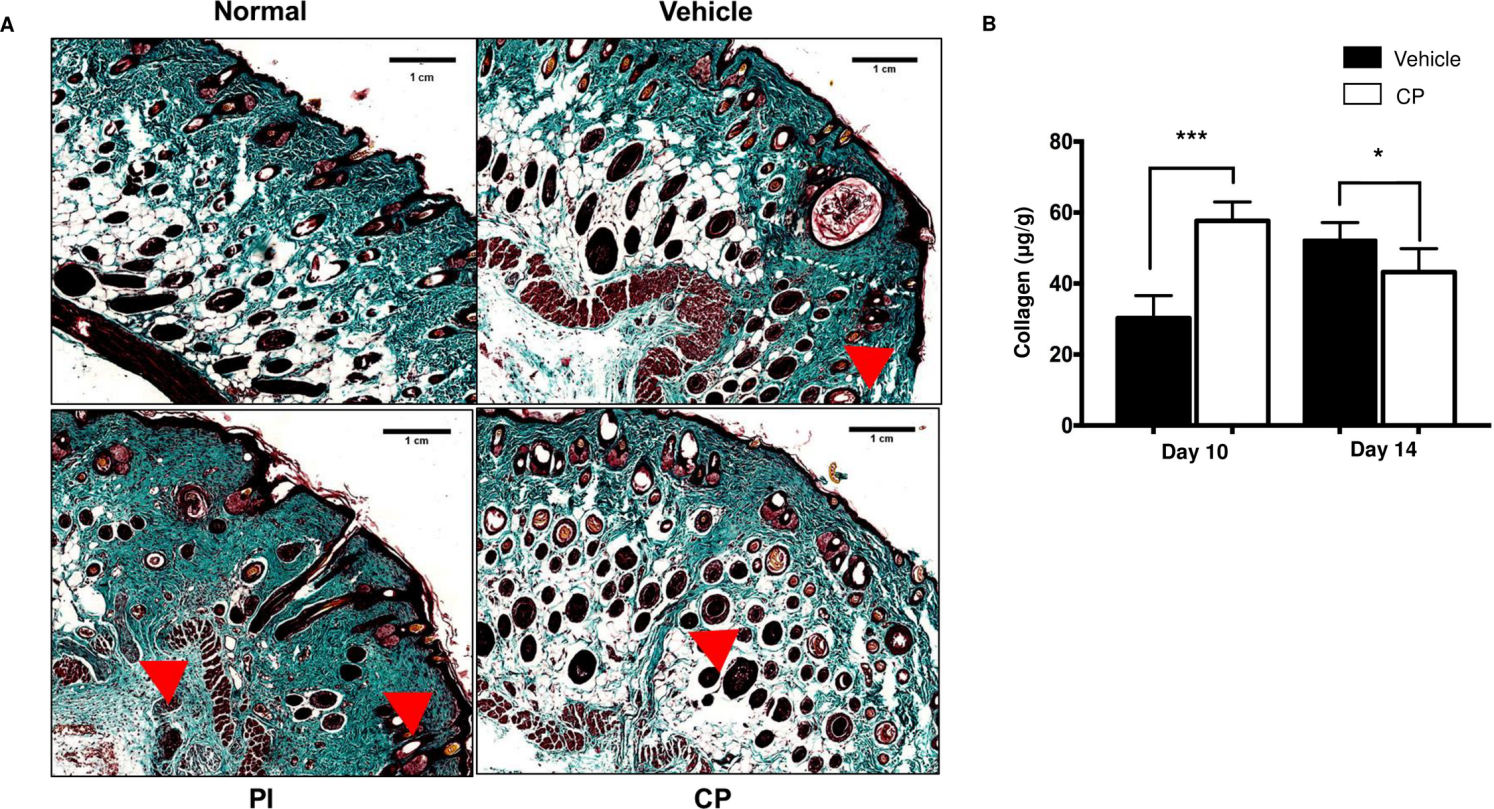
Histological and quantitative analyses of the cutaneous wound healing of calophyllolide. Mice were daily treated with CP (6 mg/animal) and PI (100 mg/animal) until enthanasia. **(A)** Histological observation of collagen on wound healing at day 14 by Masson’s Trichrome staining. Reduction of collagenous scar (arrow head) in CP-treated group compared to vehicle- and PI-treated groups. Arrows indicate wound site with scale bar = 1cm. **(B)** Representative graph of semi-quantitative collagen content at day 10 and day 14 (n = 3–4 animals per group per experiment). Data are represented as mean ± SEM and compared by one-way ANOVA.

To assess the collagenous nature of scar formation, Masson trichrome staining was performed. The CP-treated group displayed a completely reconstructed collagen deposition as compared with the control group (Fig 3A). The CP-treated group had smaller collagenous scars (triangle, Fig 3A) than vehicle- and PI-treated groups at day 14 post-wounding. Next, we measured the total soluble collagen in vehicle- and CP- treated groups by Sircol Collagen assay kit. Although there was significantly increased collagen content in wounds of the CP-treated group by up to 1.9 folds at day 10, the collagen content was reduced in the CP-treated group at day 14, when compared to the vehicle (Fig 3B).

### Characterization of spleen in wound healing

**Fig 4:**
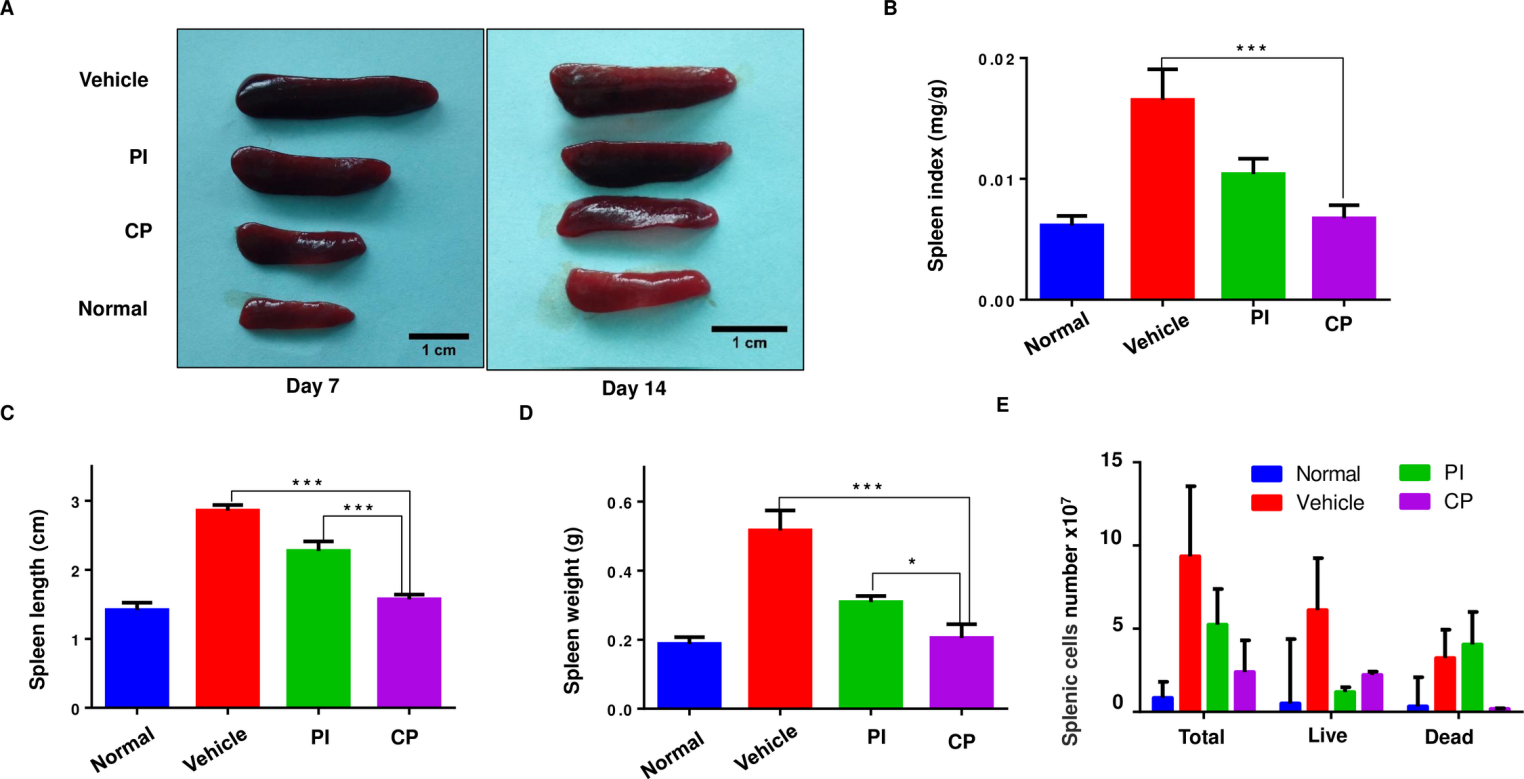
Characterization of spleen changes in wound healing. Mice were daily treated with CP (6 mg/animal) and PI (100 mg/animal) until enthanasia. **(A)** Comparison of spleen size in each group at day 7 and day 14, scale bar = 1 cm. **(B)** Spleen length, **(C)** Spleen weight, **(D)** spleen index of each group at day 7 post-operation. **(E)** Comparison of total splenic cell numbers, live and dead of splenic cells in each group at day 7. Data are represented as mean ± SEM and compared by one-way ANOVA (n = 3–4 animals per group per experiment). * P<0.05, ** P<0.01, *** P<0.001.

To test systemic effects of the wound healing process, we collected mouse spleens on day 7 and 14 post-treatment. Wounded mice treated with vehicle showed enlargement of the spleen. CP treated mice showed reduced spleen sizes compared with vehicle treated mice at day 7 and day 14 post-injury. The spleen size of CP-treated mice was restored to that of unwounded mice (control) at day 7 and 14 post-injury (Fig 4A). Splenomegaly was also decreased by treatment with PI but to a lesser extent than CP treatment. The CP-treated group demonstrated reduced spleen length/weight/index and splenic cell numbers when compared to the vehicle group at day 7 and day 14 (Fig 4B–4E and S2 Table). At day 7 and 14 post-treatment, CP reduced spleen length by 0.7 cm and 1.1 cm, and spleen weight by 0.31 g and 0.11 g as well as spleen index by 2.4 folds and 1.5 folds when compared to vehicle treated mice.

### Attenuation of inflammatory cytokines in cutaneous wound healing by calophyllolide (CP)

**Fig 5:**
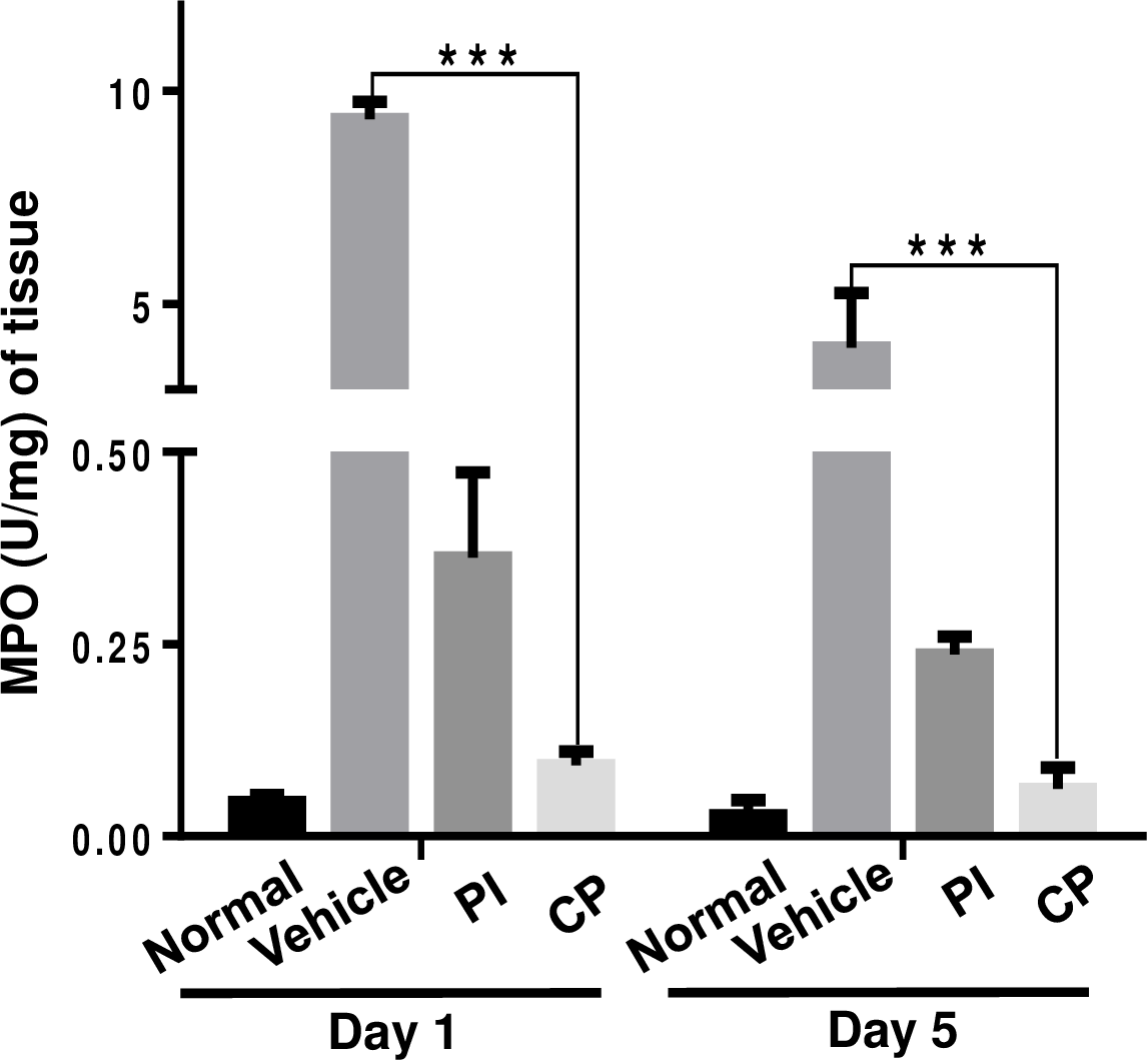
Effect of calophyllolide on myeloperoxidase (MPO) activity. All mice were sacrificed on day 1 and day 5 post-operation, and skin tissue samples were collected to assess MPO activity (n = 3 mice per group per experiment). Data are represented as mean ± SEM and compared by one-way ANOVA. *** P<0.001.

**Fig 6:**
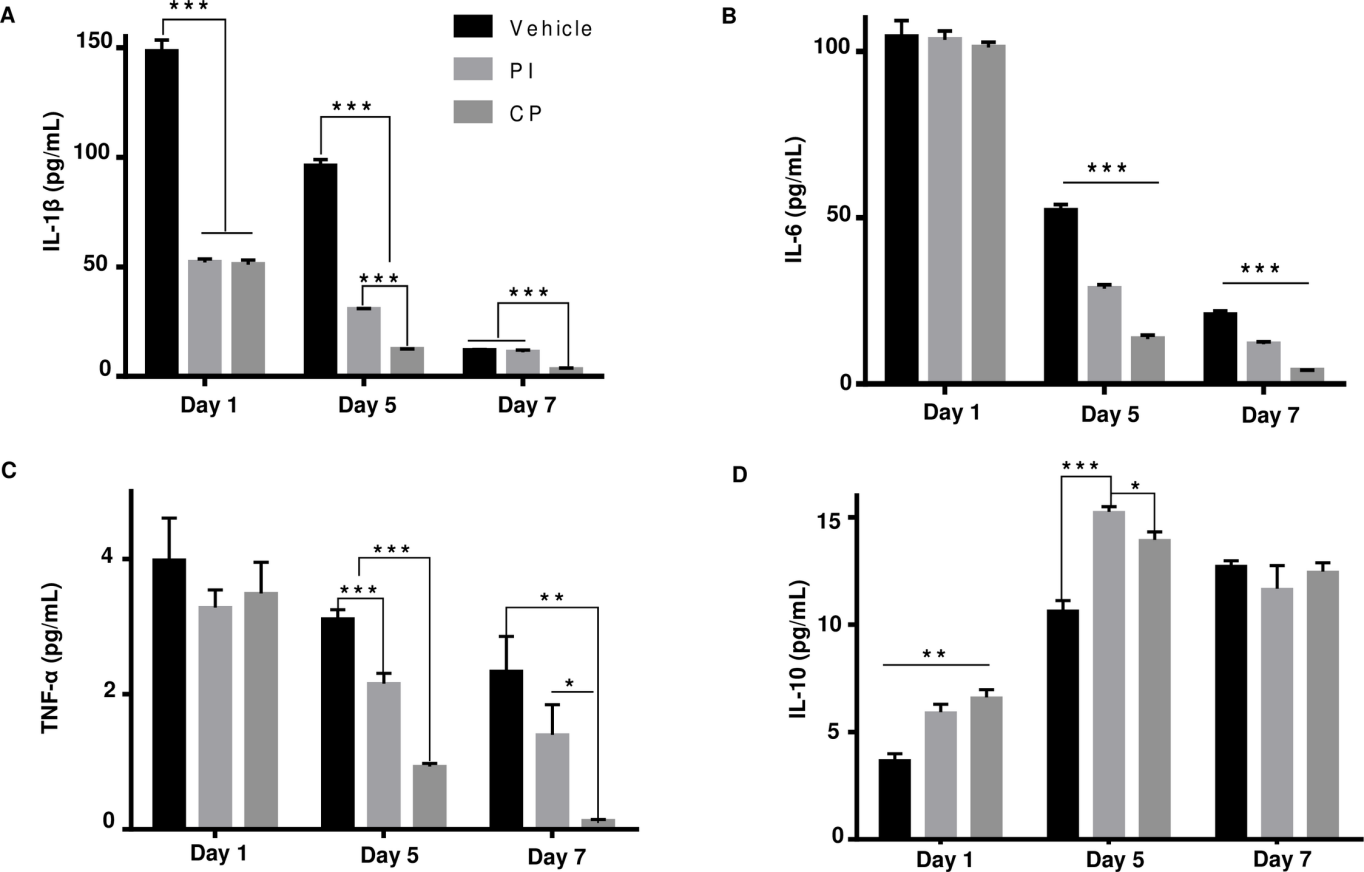
Attenuation of inflammatory cytokines expression by calophyllolide. Serum levels of (**A**) IL-1β, (**B**) IL-6, (**C**) TNF-α, and (**D**) IL-10. Data are represented as mean ± SEM and compared by one-way ANOVA (n = 3 mice per group per experiment). * P<0.05, ** P<0.01, *** P<0.001.

The anti-inflammatory activity of CP was assessed by MPO assay. When compared to vehicle (9.3 U/mg at day 1 and 4.0 U/mg at day 5), CP markedly reduced MPO activity by 9.2 U/mg (98%) at day 1 and 3.9 U/mg (97%) at day 5 post-treatment. MPO inhibition by CP was four times greater than that of PI (Fig 5). Next, we measured the expression of pro-inflammatory cytokines (IL-1β, IL-6, and TNF-α), and the anti-inflammatory cytokine (IL-10) in sera at day 1, 5 and 7 post-wounding. CP down-regulated systemic pro-inflammatory cytokines at day 5 and day 7 post-treatment by 83.7 pg/mL (87%) and 8.7 pg/mL (75%) for IL-1β, 38.5 pg/mL (74%) and 16.6 pg/mL (80%) for IL-6, 2.1 pg/mL (70%) and 2.2 pg/mL (90%) for TNF-α, when compared to vehicle, respectively (Fig 6A–6C). However, CP significantly up-regulated IL-10 anti-inflammatory cytokine expression around 3.2 pg/mL (30%) at day 5, but did not increase its expression at day 7 (Fig 6D).

### Inhibition of SLS induced inflammatory macrophages by calophyllolide (CP)

**Fig 7:**
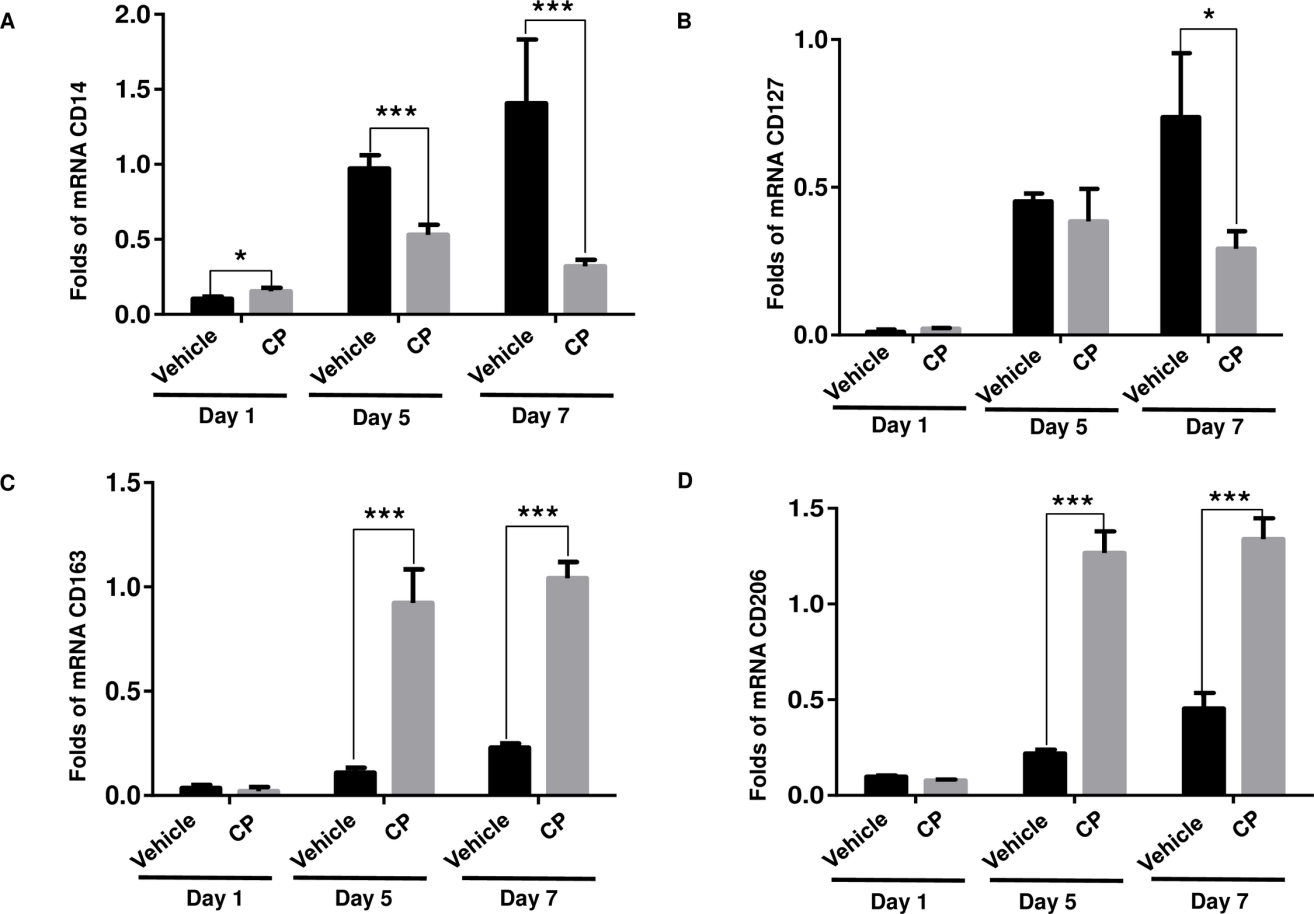
Expression of M1/M2 macrophage-related genes/markers during SLS induced inflammation. **(A and B)**: M1-related genes/markers (CD14 and CD127), **(C and D)**: M2-related genes/markers (CD163 and CD206). Expression level was measured by qRT-PCR analysis.

In order to assess the effect of CP treatment on macrophages phenotypes (M1/M2) in SLS-induced mice skin, related gene/marker expression was measured by qRT-PCR. As shown in Fig 7, CP did not affect the expression of tested genes at day 1. At day 5 and day 7, CP down-regulated M1-related gene expression (CD14 and CD127) by 1.8–4.3 folds for CD14, 1.2–2.5 folds for CD127 (Fig 7A and 7B), but up-regulated M2-related gene expression (CD163 and CD206) by 4.5–8.4 folds for CD163, 2.9–5.7 folds for CD206 at day 5 and day 7 when compared to the vehicle, respectively (Fig 7C and 7D). In other words, CP promoted the predominance of M2 macrophage cells at the site of injury.

## Discussion

Optimal topical wound treatements must be biocompatibile, nontoxic, and safe. Medicinal plant-derived compounds for enhancing cutaenous wound healing are attractive treatment options because they are both cost-effective and safe [25]. In a previous study, Leguillier *et al*. [26] demonstrated that the *C*. *inophyllum* oil could be a valuable candidate for the treatment of infected wounds due to its wound healing and antibiotic properties. The present study is the first to show that topical application of calophyllolide (CP), a major compound from *C*. *inophyllum*, can enhance cutaneous wound healing in an *in vivo* mouse model.

Calophyllolide (CP) exhibited numerous positive effects on cutaneous wound healing. CP reduced fibrosis formation and accelerated the closure of wound area with the epidermis and dermal layers completely formed by day 14 post-treatment. Body weight of CP-treated group was unaffected by administration of CP throughout the whole experimental period. Furthermore, CP treated mice showed none of the spleen enlargement noted in vehicle treated mice. Thus, CP in fact promoted wound closure without adverse, inflammatory, systemic effects.

Collagen fibers are the primary functional component of dermis tissue layer of the skin and are essential for the strength of wound (called “scarring”) which is promoted by TGF-β1 and PDGFs [27,28]. The CP-treated group displayed a completely reconstructed collagen deposition in distinguishable from the control group. CP treatment led to increased collagen synthesis at day 10, with slightly decreased formation at day 14. The underlying molecular mechanisms driving CP promotion of collagen synthesis in wound healing is as yet not understood.

Neutrophils and macrophages play key roles in the inflammatory phase of wound healing by releasing cytokines and growth factors. MPO, released from neutrophils, is part of the first line of host defense following recognition of danger signals from invading pathogens [29]. MPO is often used as the marker enzyme for the accumulation of neutrophils within skin lesions [30]. An excess of neutrophil recruitment to the wound site and excessive production of MPO will lead to elevated ROS production, thereby delaying woung healing [31]. CP reduced MPO activity in wound samples during the tested time period when compared with vehicle and PI treated mice. Moreover, during SLS-induced inflammation, CP treatment led to an up-regulation of M2-related gene expression (CD163 and CD206), and down-regulation of M1-related gene expression (CD14 and CD127). Activated macrophages can differentiate into either a classical phenotype (M1), broadly considered to be inflammatory in nature, or an alternative phenotype (M2) which enhance would healing and angiogenesis [32]. During skin wound healing a vital role is played by resident macrophages in the injury site which switch to an M2 phenotype, decreasing the inflammatory process, promoting angiogenesis, and inducing production of collagen by fibroblasts [33]. Therefore, CP treatment accelerated the tissue repair process by shifting macrophage activity to an M2 phenotype and reducing MPO activity.

The inflammatory phase is the first and essential stage in the wound healing process. However, prolonged inflammation causes enhanced release of cytokines such as IL-1β, IL-6 and TNF-α, severe healing disturbances and increased fibrosis and scarring, [1, 34, 35]. The increased and prolonged action of neutrophils and pro-inflammatory cytokines are associated with tissue damage through the production and induction of proteolytic enzymes and arachidonic acid metabolites, thereby leading to a delay in initiation of the repair phase [36]. In particular, high levels of IL-1β correlates with non-healing and scar formation [37]. Similarly, overexpression of TNF-α and IL-6 leads to destructive effects in wound healing [38], and in different pathological conditions of the skin [39]. Inhibition of these mediators may regulate the progress of cutaneous wound healing and thus represent a good therapeutic target [40]. Therefore, to understand the underlying molecular mechanisms of wound repair, we tested the effect of CP treatment on the expression of pro-inflammatory cytokines. CP treatment led to down-regulation of pro-inflammatory cytokines such as IL-1β, IL-6 and TNF-α, but up-regulation of an anti-inflammatory cytokine, IL-10. IL-10 plays an important role in the control of inflammation and immune-mediated tissue damage, and reducing the potential for the scarring [41]. One plausible explanation is that CP treatment accelerated the inflammatory process through regulation of pro-inflammatory cytokines IL-1β, IL-6 and TNF-α and anti-inflammatory cytokine IL-10. Taken together, it is clear that CP accelerates the process of wound healing through anti-inflammatory activities, namely, by regulation of inflammatory cytokines, reduction in MPO, and switching of macrophages to an M2 phenotype.

## Conclusions

This study is the first to demonstrate that calophyllolide (CP) isolated from *C*. *inophyllum* could be a good candidate for accelerating wound healing through its anti-inflammatory effects. This finding could enable the utilization of CP compound as a potent therapeutic for cutaneous wound healing treatment. Future work shall focus on preclinical investigations which could pave the way for future application of CP for cutaneous wound healing.

## Supporting information

S1 Fig ^1^H-NMR analysis of isolated calophyllolide (CP). (PDF)

S2 Fig ^13^C-NMR analysis of isolated calophyllolide (CP). (PDF)

S3 Fig LC-MS analysis of isolated calophyllolide (CP). (PDF)

S4 Fig No effect of calophyllolide on proliferation and apoptosis of wound healing. (PDF)

S1 Table List of designed forward and reverse primers for M1/M2 macrophage-related genes/markers. (PDF)

S2 Table The values of body weight and spleen from normal-, vehicle-, PI-, and CP- treated group measured at day 7 and day 14. (PDF)

S1 File BrdU and TUNEL assay. (PDF)
