# Supplementary figures and images for: Anti-inflammatory and wound healing activities of calophyllolide isolated from Calophyllum inophyllum Linn
Source: PLoS One. 2017 Oct 11;12(10):e0185674. doi: 10.1371/journal.pone.0185674 (PMC5636079; doi:10.1371/journal.pone.0185674)

S1 Fig.  $^1\text{H}$ -NMR analysis of isolated calophyllolide (CP)

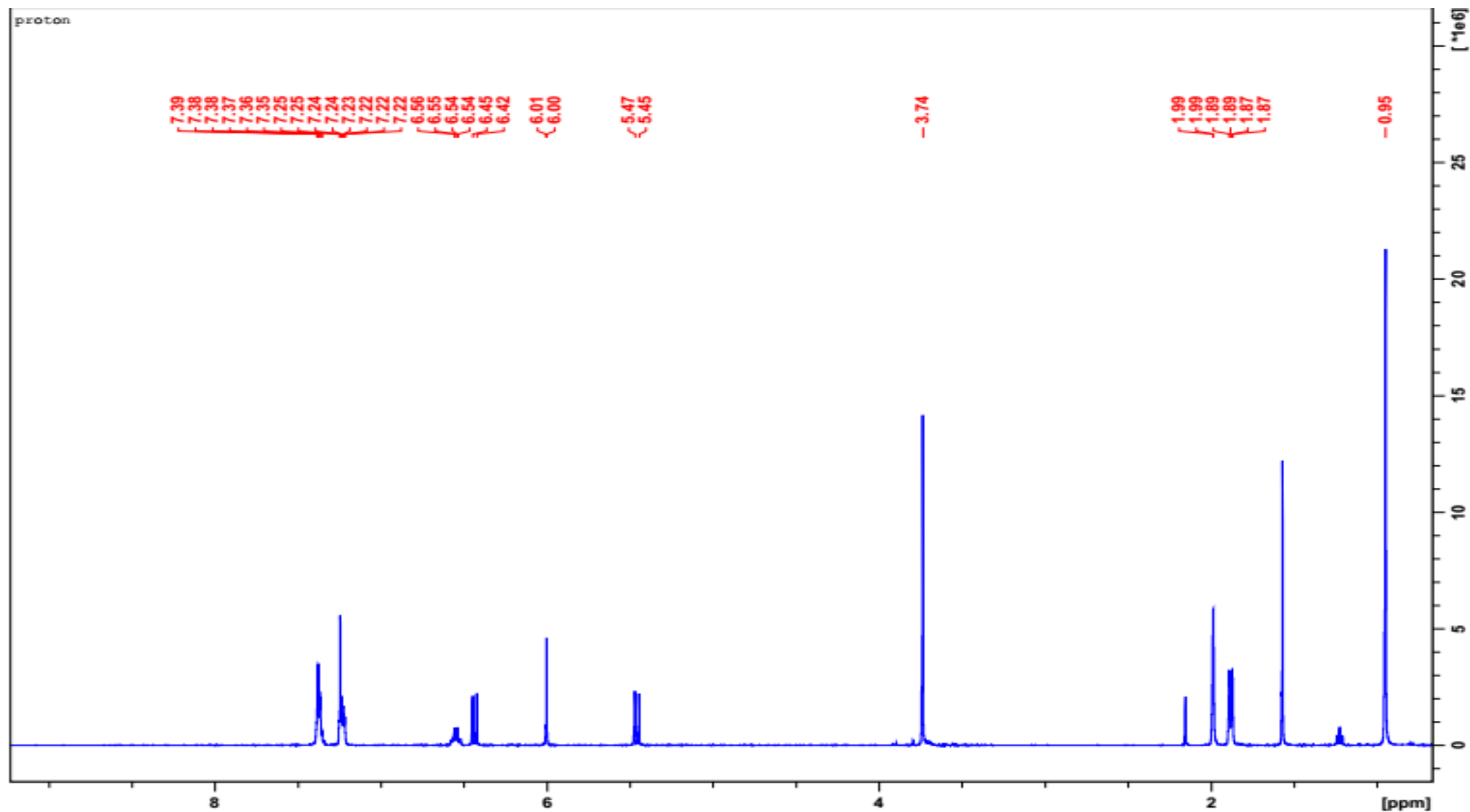

Supplement: S1 Fig — (PDF) [file pone.0185674.s001.pdf]

S2 Fig.  $^{13}\text{C}$ -NMR analysis of isolated calophyllolide (CP)

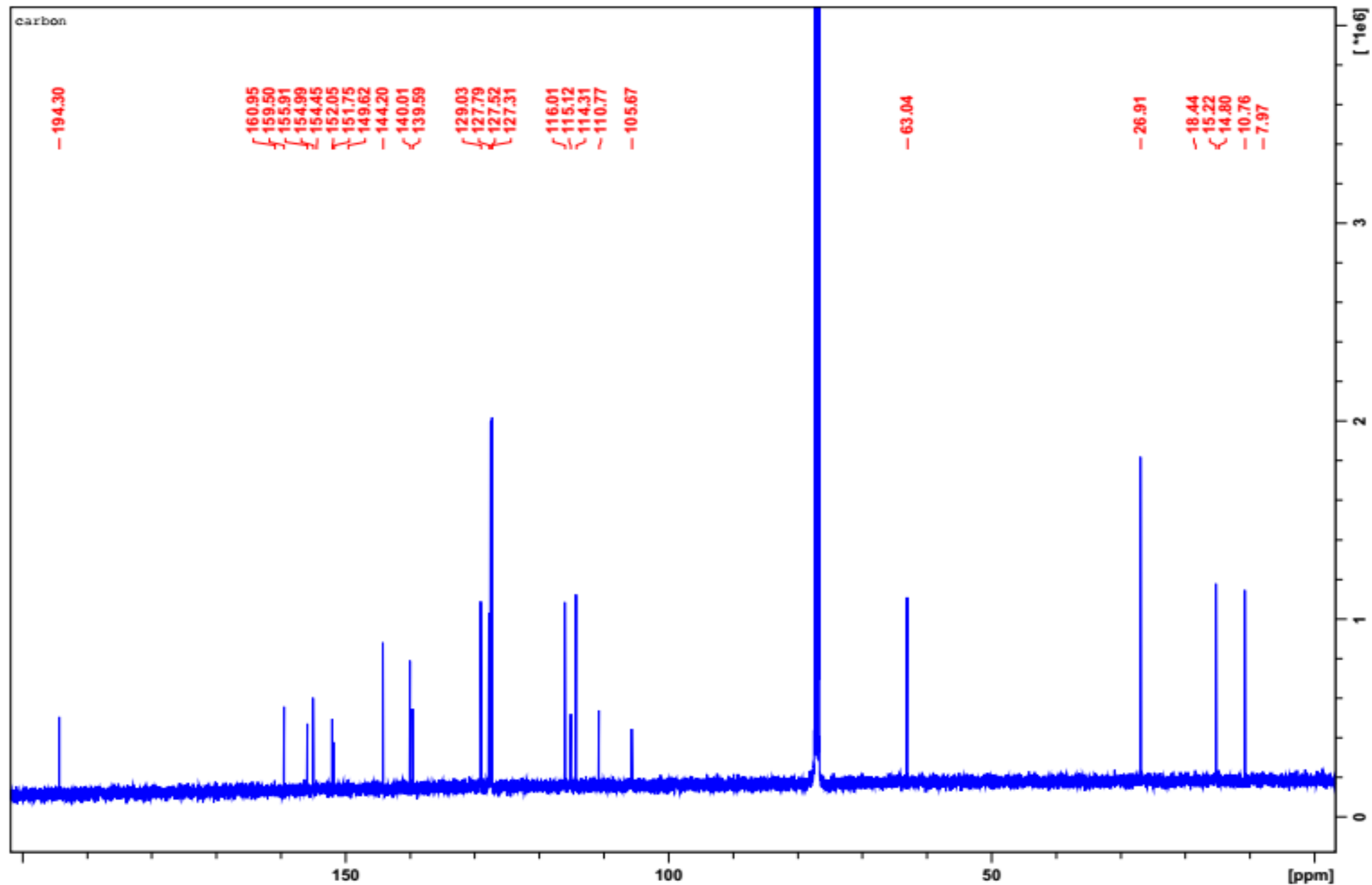

Supplement: S2 Fig — (PDF) [file pone.0185674.s002.pdf]

**S3 Fig. LC-MS analysis of isolated calophyllolide (CP)**

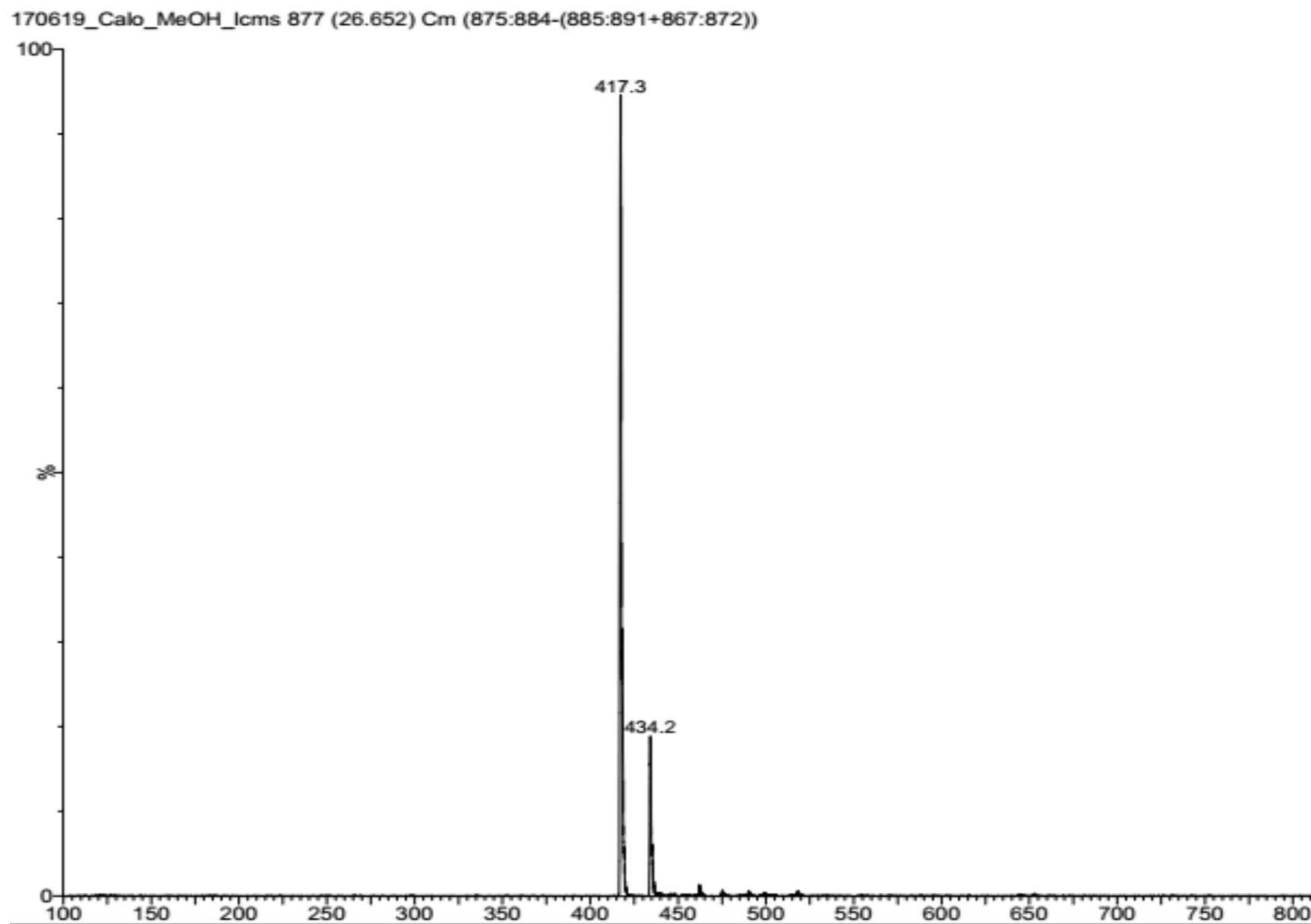

Supplement: S3 Fig — (PDF) [file pone.0185674.s003.pdf]
