# Supplementary material for: Anti-inflammatory and wound healing activities of calophyllolide isolated from Calophyllum inophyllum Linn
Source: PLoS One. 2017 Oct 11;12(10):e0185674. doi: 10.1371/journal.pone.0185674 (PMC5636079; doi:10.1371/journal.pone.0185674)
Supplement: S4 Fig — (PDF) [file pone.0185674.s004.pdf]

S4 Fig. No effect of calophyllolide on proliferation and apoptosis of wound healing

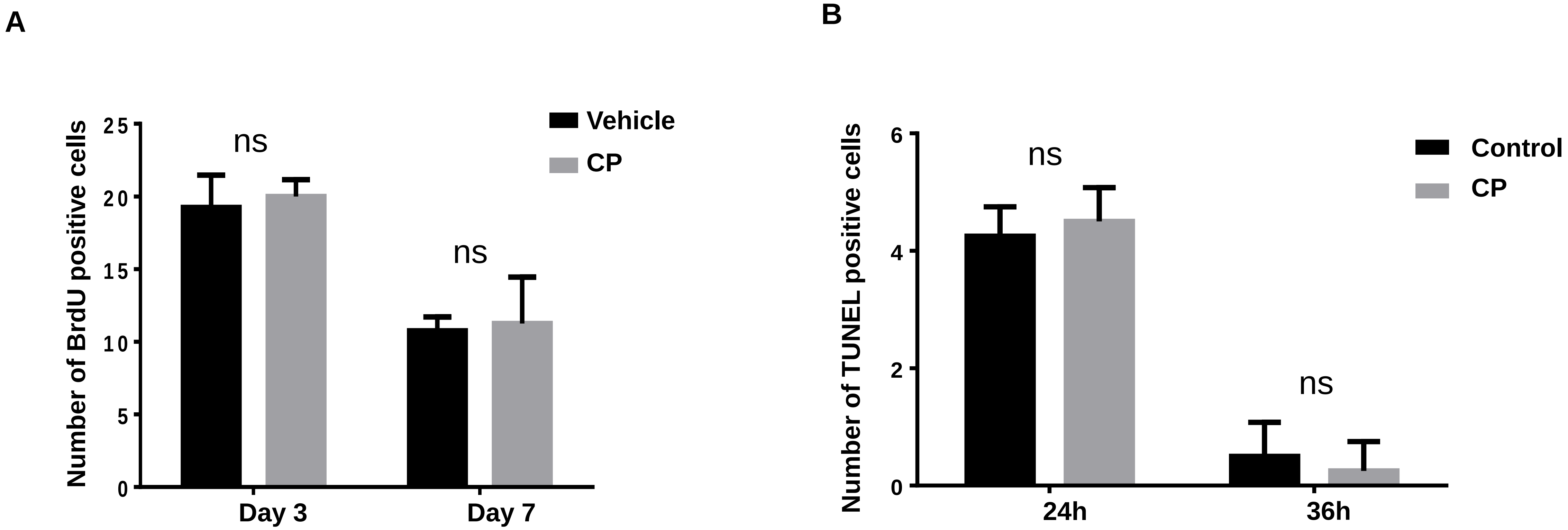

(A) Quantification of BrdU positive cells showed no significant difference between CP and vehicle group at day 3 and day 7. (B) Quantification of TUNEL staining 24 h and 36 h post-wounding showed no significant difference between CP and vehicle group. n = 3 per group. Data are represented as mean ± SD.
