# Supplementary material for: Anti-inflammatory and wound healing activities of calophyllolide isolated from Calophyllum inophyllum Linn
Source: PLoS One. 2017 Oct 11;12(10):e0185674. doi: 10.1371/journal.pone.0185674 (PMC5636079; doi:10.1371/journal.pone.0185674)
Supplement: S1 Table — (PDF) [file pone.0185674.s005.pdf]

**S1 Table. List of designed forward and reverse primers for M1/M2 macrophage-related genes/markers**

| Name | Gene Symbol | Forward primer | Reverse primer | Product (bp) |
|---|---|---|---|---|
| **M1** | CD14 | 5'-CTCAAACTTTCAGAATCTACC-3' | 5'-GACTTGATAATATCACGCAACTG-3' | 276 |
| | CD127 | 5'-TCTGACCTGAAAGTCGTTTATCGC-3' | 5'-CATCCTCCTTGATTCTTGGGTTC-3’ | 317 |
| **M2** | CD163 | 5'-AGCTGGTCAGGTCTGGAGTC -3' | 5'-CCTGTCCTTCGGAACACGTC -3’ | 107 |
| | CD206 | 5'-CGTCACCCTGTATGCCTGTG-3' | 5'-CCATCTGCTCCACAATCCCG-3' | 163 |
| **Control** | GAPDH | 5'-CCAATGTGTCCGTCGTGGATC-3' | 5'-GCTTCACCACCTTCTTGATGTC-3' | 162 |
