# Supplementary material for: Anti-inflammatory and wound healing activities of calophyllolide isolated from Calophyllum inophyllum Linn
Source: PLoS One. 2017 Oct 11;12(10):e0185674. doi: 10.1371/journal.pone.0185674 (PMC5636079; doi:10.1371/journal.pone.0185674)
Supplement: S2 Table — (PDF) [file pone.0185674.s006.pdf]

S2 Table. The values of body weight and spleen from normal-, vehicle-, PI-, and CP- treated group measured at day 7 and day 14

Parameter	Mouse group	Day	Mean	SD	P-value
Body weight (g)	Normal	7	30.50	1.229	0.6524
		14	38.48	1.337	0.4583
	Vehicle	7	31.37	1.305	0.6524
		14	37.66	1.228	0.4583
	PI	7	29.83	2.098	0.6524
		14	37.10	1.439	0.4583
	CP	7	30.30	1.054	0.6524
		14	37.78	1.305	0.4583
Spleen length (cm)	Normal	7	1.417	0.1060	0.0001
		14	1.282	0.04324	0.0001
	Vehicle	7	2.857	0.08145	0.0001
		14	2.352	0.1585	0.0001
	PI	7	2.273	0.1365	0.0001
		14	2.076	0.1234	0.0001
	CP	7	1.573	0.06807	0.0001
		14	1.304	0.07092	0.0001
Spleen weight (g)	Normal	7	0.1877	0.01943	0.0001
		14	0.2240	0.04615	0.0080
	Vehicle	7	0.5167	0.05750	0.0001
		14	0.3500	0.04472	0.0080
	PI	7	0.3090	0.01744	0.0001
		14	0.2880	0.05933	0.0080
	CP	7	0.2050	0.03969	0.0001
		14	0.2340	0.06580	0.0080
Spleen index	Normal	7	0.006166	0.0007753	0.0001
		14	0.005822	0.001179	0.0070

	Vehicle	7	0.01654	0.002529	0.0001
		14	0.009308	0.001284	0.0070
	PI	7	0.01042	0.001252	0.0001
		14	0.007777	0.001652	0.0070
	CP	7	0.006743	0.001085	0.0001
		14	0.006200	0.001742	0.0070
