# Supplementary material for: Anti-inflammatory and wound healing activities of calophyllolide isolated from Calophyllum inophyllum Linn
Source: PLoS One. 2017 Oct 11;12(10):e0185674. doi: 10.1371/journal.pone.0185674 (PMC5636079; doi:10.1371/journal.pone.0185674)
Supplement: S1 File — (PDF) [file pone.0185674.s007.pdf]

**S1 File. BrdU and TUNEL assay.** For epithelial proliferation, mice from each group were intraperitoneally injected with 40 mg/kg BrdU (GE Healthcare Life Science) 2 h prior to sacrifice following day 3 and day 7. The 10 µm cryo-sections were fixed in 4% paraformaldehyde, washed in PBS and blocked in a 5% BSA in 0.03% Triton X-100 for an hour at room temperature. Sections were then incubated with rabbit polyclonal anti-BrdU (Abcam, South Korea), followed by washing 3 times with PBS and incubatedwith Alexa Fluor® 488-conjugated secondary antibody (Thermo Fisher Scientific, South Korea). BrdU-positive cells were quantified with at least three different fields in the wound areas. The TUNEL staining was performed using an in situ cell death detection kit (Roche, South Korea) following the manufacturer's protocol at 24 h and 36 h.
